# Death of Dr. Underhill

**Published:** 1891-01

**Authors:** A. Garwood, T. J. Bennett

**Affiliations:** New Braunfels, Texas; Secretary; New Braunfels, Texas


### Society Notes.

### DEATH OF DR. UNDERHILL.

NEW BRAUNFELS, TEXAS, Dec. 17, 1890.

*Mr. President and Gentlemen Austin District Medical Society:*

It is my sad duty to announce to this Society the death of an honored member, Dr. G. Breaux Underhill. Dr. Underhill died in New Braunfels, Texas, December 10, 1890, and his remains were interred in Lafayette Cemetery, New Orleans, La., on the 12th instant.

Before offering suitable resolutions, I desire to pay humble tribute to his memory, and to express my own sorrow for the death of him, who, as a man, possessed talents and virtues worthy of emulation; who, as a friend, was warm-hearted, generous and true; who, as a physician, stood in the front rank of his profession, and, though himself diseased, and conscious of an early grave, heroically gave of his waning strength to help the feeble and comfort the poor.

The subject of this memorial was born in New Orleans, La., on the 9th of June, 1853. He inherited a nature gentle and refined, and possessed a mind too broad to harbor malice or envy for his fellow-men. As an only son, he was given every advantage of education, and in his early manhood graduated with honor at Trinity College, Hartford, Conn. His restless mental and physical energy urged him onward to the goal for which nature had best fitted him, and putting aside the temptation of a life of ease and idleness which wealth held out to him, he resumed the arduous work of student life by entering upon the study of medicine in the University of Louisiana.

Graduating in due time he at once left for Europe, and spent several months in further study at the Universities of Vienna and Heidelberg. After an absence of two years he returned to his native city and was tendered and accepted the position of consulting physician at the Charity hospital. This position he held for two years and until failing health prompted his resignation. Broken down physically, he was induced to seek our sunny climate, and with a forlorn hope he came in 1886 and spent the remainder of his days among us.

As a member of this society you each knew him only as a cheerful, earnest worker; but few knew of his heroic struggle that ended in death. Being a physician, he was painfully conscious of his certain and speedy doom; and yet, with philosophic calmness, he awaited the end.

His well-stored and disciplined mind long dominated the frail body, and a number of medical contributions from his pen affirm his intellectual energy. His papers on The Influence of Malaria on the Upper Abdominal Viscera, and Continued Fevers, read before the Society, were of more than ordinary merit and were well recieved by this body. But now his life's work is ended, and the name of G. Breaux Underhill is forever erased from the roll of the living. The records of this society prove him to have been a worthy brother while living; let these records also attest our deep sorrow that he is dead. I offer, Mr. President, the following resolutions:

*Resolved*, That the Austin District Medical Society has heard with profound sorrow of the death of Dr. G. Breaux Underhill.

*Resolved*, That this Society has lost a faithful brother and the profession a gifted son.

*Resolved*, That our sincere sympathy is with his sorrowing family.

*Resolved*, That these resolutions be spread on the minutes of this Society, and the family be furnished with a copy of the same.

A. GARWOOD, M. D.

The above memorial and resolutions were unanimously and feelingly adopted by the Society. T. J. BENNETT, Secretary.

